# High Concentration Hydrogen Protects Sepsis‐Associated Encephalopathy by Enhancing Pink1/Parkin‐Mediated Mitophagy and Inhibiting cGAS‐STING‐IRF3 Pathway

**DOI:** 10.1111/cns.70305

**Published:** 2025-02-27

**Authors:** Yan Cui, Jianfeng Liu, Yu Song, Chen Chen, Yuehao Shen, Keliang Xie

**Affiliations:** ^1^ Department of Pathogen Biology School of Basic Medical Sciences, Tianjin Medical University Tianjin China; ^2^ Department of Critical Care Medicine Tianjin Medical University General Hospital Tianjin China; ^3^ Department of Anesthesiology Tianjin Institute of Anesthesiology, Tianjin Medical University General Hospital Tianjin China

**Keywords:** cGAS‐STING‐IRF3, hydrogen, mitophagy, PINK1/Parkin, sepsis‐associated encephalopathy, tau phosphorylation

## Abstract

**Background:**

Sepsis‐associated encephalopathy (SAE) leads to increased mortality. Hydrogen (H_2_) has been proven to be effective in protecting against SAE. This study aimed to investigate the protective mechanism of a high concentration of H_2_ (HCH) (67%) against SAE.

**Methods:**

A mouse sepsis model was established via cecal ligation and puncture (CLP). 67% H_2_ was inhaled for 1 h at 1 h and 6 h after the operation. First, mice were randomly divided into 5 groups: Sham, CLP, CLP + CQ (a mitophagy inhibitor), CLP + H_2_, and CLP + H_2_ + CQ. Seven‐day survival, cognitive function, and hippocampal damage were assessed. Then, mice were randomly divided into four groups: Sham, CLP, CLP + UA (a mitophagy agonist), and CLP + H_2_. Seven‐day survival was recorded, cognitive function was assessed via Y‐maze and Morris water maze tests, and hippocampal damage was evaluated via Nissl staining. Phosphorylated tau, inflammatory factors, ATP, and antioxidant enzyme levels and mitochondrial membrane potential (MMP) were detected. Mitochondria were observed via transmission electron microscopy. The protein levels of the PINK1/Parkin pathway and STING‐TBK‐IRF3 pathway were detected via western blotting.

**Results:**

HCH inhalation improves 7‐day survival and cognitive function in septic mice and reduces brain tissue damage, proinflammatory cytokine levels, and phosphorylated tau levels. These effects were reversed by a mitophagy inhibitor. HCH significantly improves mitochondrial function, enhances PINK1/Parkin‐mediated mitophagy, and reduces the activity of the STING‐TBK‐IRF3 pathway in brain tissue.

**Conclusions:**

HCH inhalation effectively improved the survival rate of septic mice, alleviated SAE, and reduced tau phosphorylation. The mechanism may involve HCH enhancing PINK1/Parkin‐mediated mitophagy, which inhibits the activity of the cGAS‐STING‐IRF3 pathway, thereby reducing neuroinflammation.

## Graphical abstract

Inhalation of 67% hydrogen gas exerts a protective effect on sepsis‐associated encephalopathy, effectively improving the survival rate of septic mice, protecting cognitive function, and reducing tau protein phosphorylation. The mechanism may involve hydrogen enhancing PINK1/Parkin‐mediated mitophagy, which inhibits the activity of the cGAS‐STING‐IRF3 pathway, thereby reducing neuroinflammation.

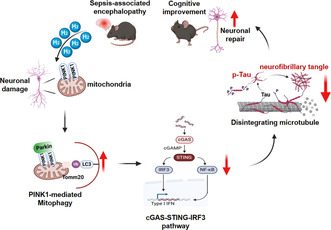

**Abbreviations**
cGAMP: cyclic GMP‐AMP
cGAS: cyclic GMP‐AMP synthase
CLP: cecal ligation and puncture
CQ: chloroquine
DMSO: dimethyl sulfoxide
ELISA: enzyme‐linked immunosorbent assay
H_2_
: hydrogen
HCH: high concentration of H_2_
IFN: type 1 interferon
IRF3: interferon regulatory factor 3
MMP: mitochondrial membrane potential
MSS: murine sepsis score
mtDNA: mitochondrial DNA
MWM: Morris water maze
PINK1: PTEN‐induced kinase 1
SAE: sepsis‐associated encephalopathy
SAP: spontaneous alternation performance
SOD: superoxide dismutase
STING: stimulator of interferon genes
TBK1: TANK‐binding kinase 1
UA: urolithin A

## 1 Introduction

With the increase in life expectancy and the intensification of population aging worldwide, the incidence of sepsis is increasing annually. Although the acute mortality rate of sepsis has declined with improvements in diagnosis and treatment methods, the long‐term mortality rate of surviving patients has increased significantly, resulting in long‐term complications, which significantly affect quality of life and cause heavy economic and care burdens for society and families [1]. Sepsis‐associated encephalopathy (SAE) is one of the most common complications in sepsis patients and significantly increases the acute and long‐term mortality rates of patients. In addition to alterations in mental status during the acute stage, approximately half of sepsis survivors still have cognitive dysfunction, such as impaired attention and memory, 1 year after discharge, which seriously affects their quality of life and significantly increases the risk of long‐term dementia [2, 3].

The pathophysiological mechanism of SAE is complex and still unclear, which may be related to oxidative stress, elevated cytokines, etc. Oxidative stress can lead to abnormal neural signaling and neuronal apoptosis in SAE, causing cognitive dysfunction [3]. Hydrogen (H_2_), as a new medical gas that can penetrate the blood–brain barrier, has been proven to have significant antioxidant effects [4, 5]. Our previous studies confirmed that a low concentration of H_2_ (2%) can effectively improve acute and long‐term survival rates and mitigate cognitive dysfunction in SAE mice. The mechanism is closely related to reducing damage to hippocampal tissue and protecting the structure and function of dendritic spines [6, 7, 8]. Treatment with 2% H_2_ significantly improved postoperative cognitive function and delirium in elderly patients undergoing surgery [9]. However, the preparation and storage of low‐concentration H_2_ have certain safety risks in daily practice, which limits the clinical application of H_2_. In contrast, water electrolysis can obtain H_2_ at a concentration of 67%, which is the most convenient and safest method for H_2_ production at present, and recent studies have shown that this high concentration of H_2_ (HCH) has a good therapeutic effect on a variety of diseases [10, 11]. Our previous research revealed that HCH has a significant protective effect on SAE [12], but its mechanism is still unclear.

Many studies have reported that abnormal phosphorylation of the tau protein is closely related to cognitive impairment in several acute and chronic diseases, such as SAE [13, 14, 15, 16, 17], and impaired mitophagy in neurons is a key mechanism for abnormal phosphorylation of the tau protein [18, 19, 20]. Mitochondria provide more than 90% of the energy needed by the brain and play a critical role in its normal function. Mitophagy targets and degrades severely damaged mitochondria and is crucial for their function and repair. Several studies have revealed that mitophagy dysfunction plays a central role in the cognitive impairments associated with various central nervous system diseases [21, 22]. The mechanism may be related to oxidative stress damage caused by ROS accumulation, neuroinflammation caused by the release of mitochondrial damage‐related molecules, and the hyperphosphorylation of tau protein caused by PTEN‐induced kinase 1 (PINK1)‐dependent pathways [18, 23]. Among them, PINK1 precisely regulates mitophagy through a ubiquitin‐dependent pathway and plays an important neuroprotective role in a variety of acute and chronic central nervous system diseases [24, 25, 26]. The PINK1 pathway plays a core role in mitophagy, regulating tau protein phosphorylation [18, 19].

In addition, cyclic GMP‐AMP synthase (cGAS), which senses double‐stranded DNA in cells, can activate stimulator of interferon genes (STING), which binds to TANK‐binding kinase 1 (TBK1), causing its phosphorylation and the activation of interferon regulatory factor 3 (IRF3), which leads to the secretion of inflammatory cytokines such as TNF‐α [27, 28, 29]. Abnormal activation of STING is associated with multiple organ damage during sepsis, so blocking this activation is highly important for the treatment of sepsis [30, 31]. Effective mitophagy can not only prevent excessive accumulation of mitochondrial DNA (mtDNA) but also maintain homeostasis of the nervous system by inhibiting the activation of the STING‐TBK1‐IRF3 pathway [32, 33]. Our previous studies revealed that mitophagy dysfunction plays an important role in SAE‐related cognitive impairments, but the underlying mechanism has not yet been fully clarified [6, 7].

Therefore, the purpose of this study was to verify the protective effect of HCH inhalation on SAE and its effect on tau protein phosphorylation and to explore whether this protective effect is related to the regulation of mitophagy, the regulatory effect of HCH on the PINK1/Parkin pathway, and the regulation of the cGAS‐STING‐IRF3 pathway.

## 2 Materials and Methods

### 2.1 Animals

Male C57BL/6J mice (6–8 weeks, 20–25 g) were obtained from the Laboratory Animal Center of the Chinese Academy of Medical Sciences, Beijing, China. The mice had 7 days to acclimate before the experiment. All the mice were kept in a pathogen‐free environment at a temperature of 20°C–22°C and a humidity of 50%–60%, and they could freely access food and water. The Tianjin Medical University Experimental Animal Management Committee approved all the experimental protocols [IRB2022‐DWFL‐587], and all the animal studies were conducted in accordance with the ARRIVE guidelines.

### 2.2 Cecal Ligation and Puncture (CLP)

A sepsis model was constructed via cecal ligation and puncture. After 2% sevoflurane anesthesia, a longitudinal incision of 0.8 cm was made on the abdomen. The cecum was ligated with surgical sutures at the end of the 1/2 distance below the ileocecal flap and then punctured with a 21 G sterile needle. After some of the intestinal contents were squeezed out, the cecum was placed back into the abdominal cavity, and then the peritoneum and skin were closed by suturing. A single dose of sterile saline preheated at 37°C (50 mL/kg) was injected subcutaneously immediately after surgery. The abdominal wound was treated with lidocaine ointment for analgesia, which was repeated every 6 h for 2 consecutive days. In addition, meropenem (20 mg/kg in 200 μL of sterile saline) was intraperitoneally injected 6 h after surgery.

### 2.3 Experimental Procedures

First, the mice were randomly assigned to five groups (*n* = 20 per group): Sham, CLP, CLP treated with chloroquine (CQ, a mitophagy inhibitor) (CLP + CQ), CLP treated with 67% H_2_ (CLP + H_2_), and CLP treated with CQ and 67% H_2_ (CLP + H_2_ + CQ). In the Sham group, only laparotomy without ligation of the caecal puncture was performed. The sepsis model was induced via the CLP procedure in all the other four groups. The CLP + H_2_ and CLP + H_2_ + CQ groups inhaled 67% H_2_ for one hour at 1 h and 6 h after CLP surgery, whereas the other groups inhaled only air. The CLP + CQ group and the CLP + H_2_ + CQ group were treated with CQ, purchased from Glpbio, dissolved in dimethyl sulfoxide (DMSO, Sigma, USA), and injected intraperitoneally (5 mg/kg) immediately after surgery. The survival rate (*n* = 20) was monitored 7 days after surgery. Six mice were randomly selected from each group 24 h after surgery for the evaluation of hippocampal damage. The Morris water maze test was conducted to evaluate cognitive function (*n* = 8).

Next, the mice were randomly assigned to four groups: Sham, CLP, CLP treated with urolithin A (UA, a mitophagy agonist) (CLP + UA), and CLP treated with 67% H_2_ (CLP + H_2_). The CLP + H_2_ group inhaled 67% H_2_ for one hour at 1 h and 6 h after surgery, whereas the other groups inhaled only air. The CLP + UA group was treated with UA, an intestinal microbial metabolite of ellagic acid with anti‐inflammatory, anti‐proliferative, and antioxidant properties, purchased from Molbase and dissolved in DMSO (Sigma, USA) and injected intraperitoneally for 7 days. The survival rate (*n* = 20) was monitored 7 days after surgery. Six mice were randomly selected from each group 24 h after surgery for the evaluation of hippocampal damage, and their serum was collected for inflammatory factor detection. The hippocampi of 30 mice were collected to detect ATP content (*n* = 6), the mitochondrial membrane potential (*n* = 6), antioxidant enzyme activity (*n* = 6), mitochondrial morphology (*n* = 6) and Western blotting (*n* = 6). The Y maze (*n* = 8) and Morris water maze test (*n* = 8) were conducted on postoperative Days 7 and 8, respectively.

### 2.4 Hydrogen Gas Treatment

The mice in the CLP + H_2_ group and CLP + H_2_ + CQ group were placed in sealed plastic boxes with both inflow and outflow outlets. An AMS‐H‐01 hydrogen gas and oxygen atomizer (Shanghai Asclepius Meditec Co., Shanghai, China) was used to produce a mixture of 67% hydrogen and 33% oxygen, and the H_2_ concentration was continuously monitored via a heat tracing gas analyzer (Thermal Fisher, Waltham, MA, USA). Before the experiment, the box was filled with the hydrogen–oxygen mixture for 30 min. The mice inhaled the appropriate concentration of H_2_ at 1 h and 6 h after surgery. The mice in the other groups were placed into another box identical to the one above and breathed air.

### 2.5 Morris Water Maze (MWM) Test

The MWM experiment was conducted in a circular pool (120 cm in diameter and 30 cm in height) at a water temperature of 23°C ± 1°C, and a suitable amount of titanium dioxide was added to make the water opaque. The pool was divided into four quadrants, and a circular platform with a diameter of 6 cm was placed in the target quadrant. During the first 4 days, each mouse was placed into the water from four different quadrants facing the pool wall, allowing them to freely find the platform. If the mouse could not find the platform within 60 s, it was manually guided to the platform to rest for 10 s. The latency periods of each mouse were calculated by averaging the latency period of the four quadrants. On the 5thth day, the platform was removed, the mice were allowed to swim freely for 60 s, and the number of platform crossings and the residence time in the target quadrant were recorded.

### 2.6 Y Maze Test

The Y maze spontaneous alternation performance (SAP) test was performed on the 7th days after surgery to measure the ability to recognize previously explored environments. The Y maze (Shanghai Xinsoft Information Technology Co.) consisted of three arms (8 × 30 × 15 cm), with an angle of 120° between each other. The number of entries and alterations was recorded by the ANY‐maze video tracking system. The mice were introduced to the center of the Y maze and left to explore the maze for 10 min. Between trials, the arms were cleaned with 75% ethanol to eliminate residual odors. SAP is the subsequent entry into a novel arm over the course of three entries. The percentage of SAP is calculated by the number of actual alternations/(total arm entries −2) × 100.

### 2.7 Brain Histopathological Examination

The brain tissues of the mice were removed, fixed in 4% paraformaldehyde overnight at 4°C, fixed in 10% formalin for 24 h, and embedded in paraffin. The 10 μm sections were dewaxed and rehydrated with xylene and ethanol, and Nissl staining was performed. The neuronal organization and niche morphology of the CA1 hippocampus were carefully examined under a light microscope (Olympus, Japan). Three sections were randomly selected from each sample, and three fields of view of each section were observed by two pathologists who were unaware of the experiment.

### 2.8 Enzyme‐Linked Immunosorbent Assay (ELISA)

Twenty‐four hours after modeling, the hippocampus was weighed, and 90 μL of PBS was added to every 10 mg of brain tissue for grinding. The samples were centrifuged at 4000 *g* for 10 min, and the serum was collected. ELISA was used to measure the serum concentrations of TNF‐α (MTA00B, R&D Systems, USA), IL‐1β (MLB00C, R&D Systems, USA), and IL‐6 (mI098430, mlbio, China).

### 2.9 Antioxidant Enzyme Activity Detection

Twenty‐four hours after modeling, the hippocampal tissue of the mice was extracted. The activity of superoxide dismutase (SOD) was tested via a 722 visible spectrophotometer (Shanghai Analytical Instrument Factory) with Cayman kits obtained from the U.S.A.

### 2.10 JC‐1 Assay of the Mitochondrial Membrane Potential (MMP)

The hippocampus was mixed well with mitochondrial isolation solution A. The homogenate was centrifuged at 1000 *g* for 10 min at 4°C, and the supernatant was discarded. Mitochondria were collected from the precipitate by centrifugation at 11,000 *g* for 10 min at 4°C. Preservation solution (400 μL/g tissue) was added to measure the protein concentration, and the MMP was measured according to the red and green fluorescence intensities. JC‐1 staining solution (0.9 mL) was mixed with the mitochondria, and excitation wavelengths of 490 nm and 525 nm were measured via the EnSpire multifunctional enzyme marker (PerkinElmer, USA). The emission wavelengths were 530 nm and 590 nm.

### 2.11 ATP Content Measurement

Hippocampal tissue was placed in ATP detection buffer (Biyuntian Institute of Biotechnology) and homogenized 10 times with a precooling glass homogenizer. The homogenate was centrifuged at 12,000 *g* for 5 min at 4°C, and the supernatant was discarded. A 20 μL tissue sample was mixed with the EnSpire multifunctional enzyme marker (PerkinElmer, USA) to measure the relative para‐optical unit values in the assay wells, and the resulting standard curve was used to calculate the ATP content.

### 2.12 Western Blotting

Hippocampal tissue was weighed, placed in precooled PBS buffer containing protease inhibitors, homogenized for 5 min via an ultrasonic cell crusher, and centrifuged at 15000 *g* at 4°C for 10 min. The supernatant was subjected to BCA protein quantification, added to protein loading buffer, boiled, denatured, and stored at −80°C. After electrophoretic separation and membrane transfer, the membrane was blocked with skim milk and incubated with primary antibodies against PINK1 (1:2000, Santa Cruz, USA), Parkin (1:1000, Abcam, USA), AT8 (1:1000, Abcam, USA), T22 (1:1000, Sigma–Aldrich, USA), p‐Ser422 (1:1000, Abcam, USA), total tau (1:1000, Abcam, USA), and GAPDH (1:10,000, Abcam, USA) overnight at 4°C. Then, the samples were washed with TBST for 5 min and incubated with a goat anti‐rabbit secondary antibody (1:5000, Affinity, Australia) at room temperature for 1 h. ImageJ software was used to analyze the gray value, and the expression level was calculated as the ratio of the gray value of the target protein to that of the reference GAPDH band.

### 2.13 Immunofluorescence

Mouse brain tissue was placed in 4% paraformaldehyde overnight at 4°C. After dehydration, OCT reagent (4593, Solarbio, China) was used for embedding, and the embedded blocks were cut into 10 μm slices. The primary antibody PINK1 (1:200, sc‐517,353, Santa Cruz, USA) was added to the sections separately overnight at 4°C. The next day, a fluorescent secondary antibody was added to the sections, and the sections were observed under a fluorescence microscope (Olympus, Japan).

### 2.14 Statistical Analysis

Statistical analysis was performed via *SPSS* 27.0 software. The Shapiro–Wilk test was used to evaluate normality, and Levene's test was used to evaluate the homogeneity of variances. The data conformed to normal distribution and the variance was homogeneous. Values were expressed as percentages for survival rates or the mean ± SEM. *T*‐test was used for comparison between two groups, and one‐way ANOVA was used for comparison between multiple groups. Tukey's test was adopted as a post hoc test. The survival rates of animal models were compared by the log‐rank test. For all analyses, *p* < 0.05 was considered statistically significant.

## 3 Results

### 3.1 HCH Inhalation Improved 7‐Day Survival, Cognitive Function, and Brain Damage of Septic Mice, and These Effects Were Reversed by a Mitophagy Inhibitor

**FIGURE 1:**
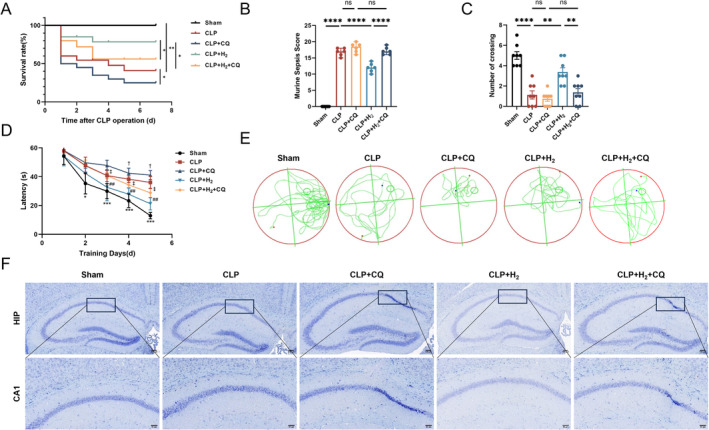
The therapeutic effect of 67% H_2_ inhalation on SAE is inhibited by a mitophagy inhibitor. (A) 7‐day survival rate of mice (*n* = 20); (B) Murine Sepsis Score (*n* = 6); (C) The number of times the mice crossed the platform in MWM test on 5th day after training (*n* = 8); (D) Escape latency during training in MWM test (*n* = 8). *p < 0.05, ***p < 0.001, Sham versus CLP; ^##^ *p* < 0.01, CLP versus CLP + H_2_; ^†^ *p* < 0.05, CLP versus CLP + CQ; ^‡^ *p* < 0.05, CLP + H_2_ versus CLP + H_2_ + CQ; (E) Trajectory plot of the 5th day after training (F) Nissl staining of hippocampus (×200). Log‐rank test was used in (A), one‐way ANOVA with Tukey's post hoc test was used in (B–D). Results are expressed as means ± SEM; *p < 0.05, **p < 0.01, ***p < 0.001, ****p < 0.0001.

As shown in Figure 1A–F and Figure S1A,B, compared with those of the CLP group, the 7‐day survival rate of the CLP + H_2_ group was greater, the murine sepsis score (MSS) was lower, the latency in the MWM test was shorter, the number of crossings was greater, and the number of normal neurons in the hippocampus was greater according to Nissl staining; these differences were all statistically significant. These findings suggest that HCH inhalation can significantly improve the 7‐day survival rate, cognitive function, and brain tissue damage in septic mice. Compared with the CLP group, the CLP + CQ group presented a lower 7‐day survival rate, greater MSS, poorer cognitive function, and more severe brain tissue damage. More importantly, mice in the CLP + H_2_ + CQ group presented changes similar to those in the CLP + CQ group, indicating that the protective effect of HCH was reversed after the addition of the mitophagy inhibitor.

### 3.2 HCH Inhalation Improved the MSS Score and 7‐Day Survival Rate of Septic Mice and Alleviated Their Cognitive Dysfunction

**FIGURE 2:**
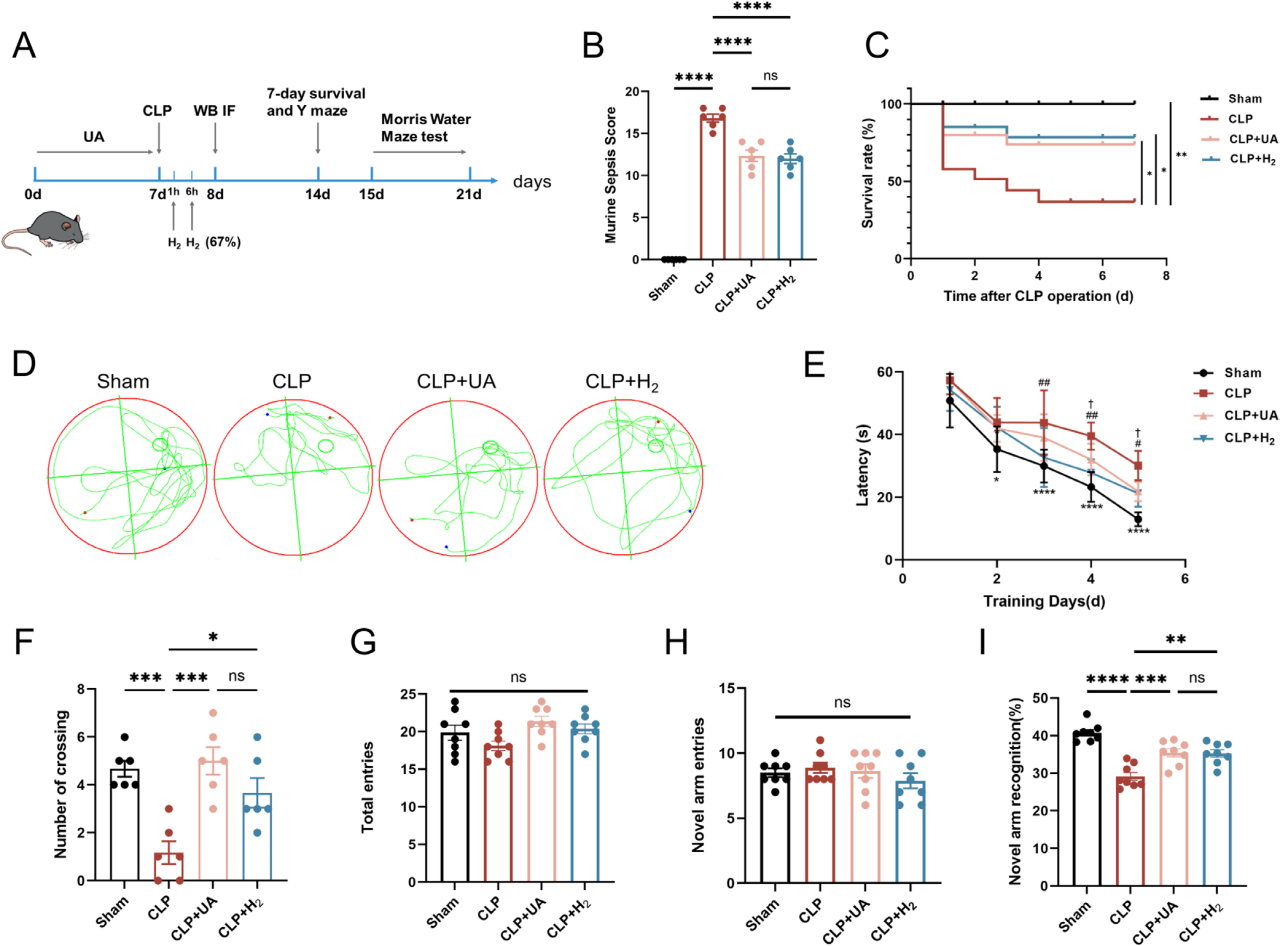
H_2_ inhalation improved MSS score and 7‐day survival of septic mice, and alleviated cognitive dysfunction. (A) Experimental flow chart; (B) Murine sepsis score (*n* = 6); (C) 7‐day survival rate (*n* = 20); The MWM test (D–F, *n* = 8). (D) Trajectory plot of the 5th day after training; (E) Escape latency during training. *p < 0.05, ****p < 0.0001, Sham versus CLP; ^#^ *p* < 0.05, ^##^ *p* < 0.01, CLP versus CLP + H_2_; ^†^ *p* < 0.05, CLP versus CLP + UA; (F) The number of times the mice crossed the platform on the 5th day after training; Y‐maze test (G–I, *n* = 8). (G) The total distance traveled; (E) The number of times the new arm was entered; (F) Percentage of time spent exploring the new arm. Log‐rank test was used in (C), one‐way ANOVA with Tukey's post hoc test was used in (B, E–I). Results are expressed as means ± SEM; *p < 0.05, **p < 0.01, ***p < 0.001, ****p < 0.0001.

**FIGURE 3:**
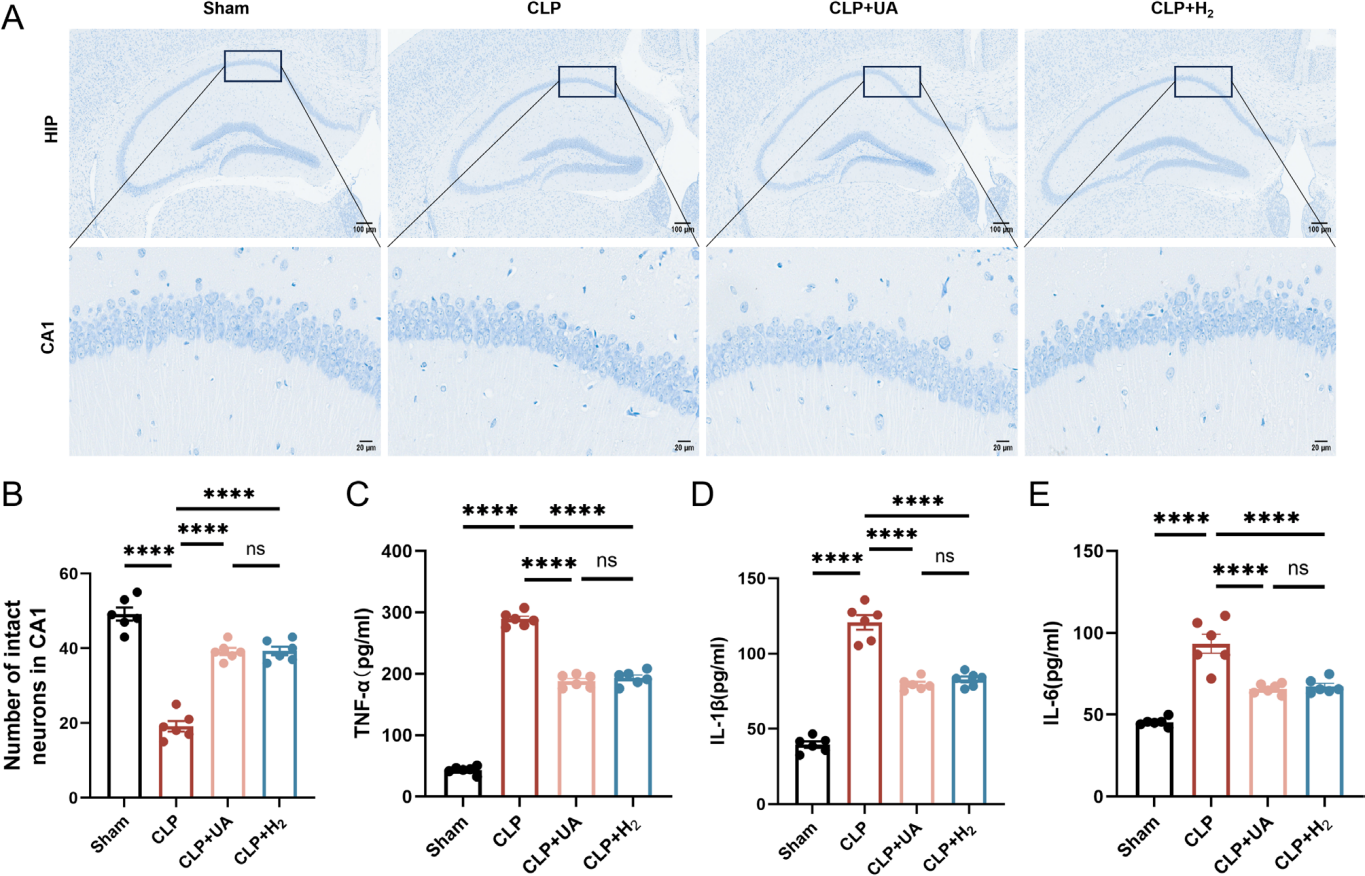
67% H_2_ inhalation reduced hippocampal tissue damage in septic mice. (A) Nissl staining of hippocampus (×200); (B) The number of intact neurons in the CA1 region was calculated via Nissl staining; (C–E) TNF‐α, IL‐1β, and IL‐6 levels in hippocampal tissue. *p* values were calculated using one‐way ANOVA followed by Tukey's post hoc test. Results are expressed as means ± SEM; *n* = 6; ****p < 0.0001.

Compared with that of the Sham group, the MSS of the CLP group was greater, but it was lower after treatment with HCH or UA (Figure 2B). The 7‐day survival rate of the mice in the CLP group was 50%, which increased to 70% in both the CLP + H_2_ and CLP + UA groups (Figure 2C). The MWM test results (Figure 3D–F, Figure S1C,D) revealed that within the same training time, the mice in the CLP group spent more time searching for the platform than those in the Sham group did, which was significantly shorter after treatment with HCH or UA (Figure 3E). After the platform was removed, the number of times the mice crossed the platform in the CLP group was lower than that in the Sham group, whereas it was greater in the CLP + H_2_ and CLP + UA groups (*p* < 0.01), with no significant difference between these two groups (Figure 3F). The Y maze test (Figure 3G–I) revealed no significant difference in the motor ability of the mice among all the groups (Figure 3G,H). The new arm exploration time of the CLP group was shorter than that of the Sham group, which was significantly longer after treatment with HCH or UA (*p* < 0.01) (Figure 3I). These results indicate that HCH inhalation protects the survival rate and MSS score of septic mice and improves their cognitive function, suggesting that HCH plays a protective role similar to that of mitophagy agonists.

### 3.3 HCH Inhalation Significantly Improved Hippocampal Tissue Damage in Septic Mice, Exerting Effects Similar to Those of Mitophagy Agonists

Neuronal cells in the hippocampal CA1 region of the mice in the Sham group were arranged neatly, and numerous Nissl vesicles were observed inside the neurons. The arrangement of neurons in the CLP group was sparse, and the number of Nissl‐stained vesicles decreased. Compared with those in the CLP group, the morphology and structure of the neurons in the CLP + H_2_ and CLP + UA groups were improved, the number of pathological changes was reduced, and the number of intact neurons in CA1 was significantly increased (*p* < 0.0001) (Figure 3A,B). The expression levels of the inflammatory factors TNF‐α, IL‐1β and IL‐6 in brain tissue were detected (Figure 3C–E) and were significantly greater in the CLP group than in the Sham group but significantly lower in the CLP + H_2_ group and CLP + UA group than in the CLP group (*p* < 0.0001).

### 3.4 HCH Inhalation Reduced Tau Phosphorylation at Key Sites in the Hippocampal Tissue of Septic Mice and Protected Neurocognitive Function

**FIGURE 4:**
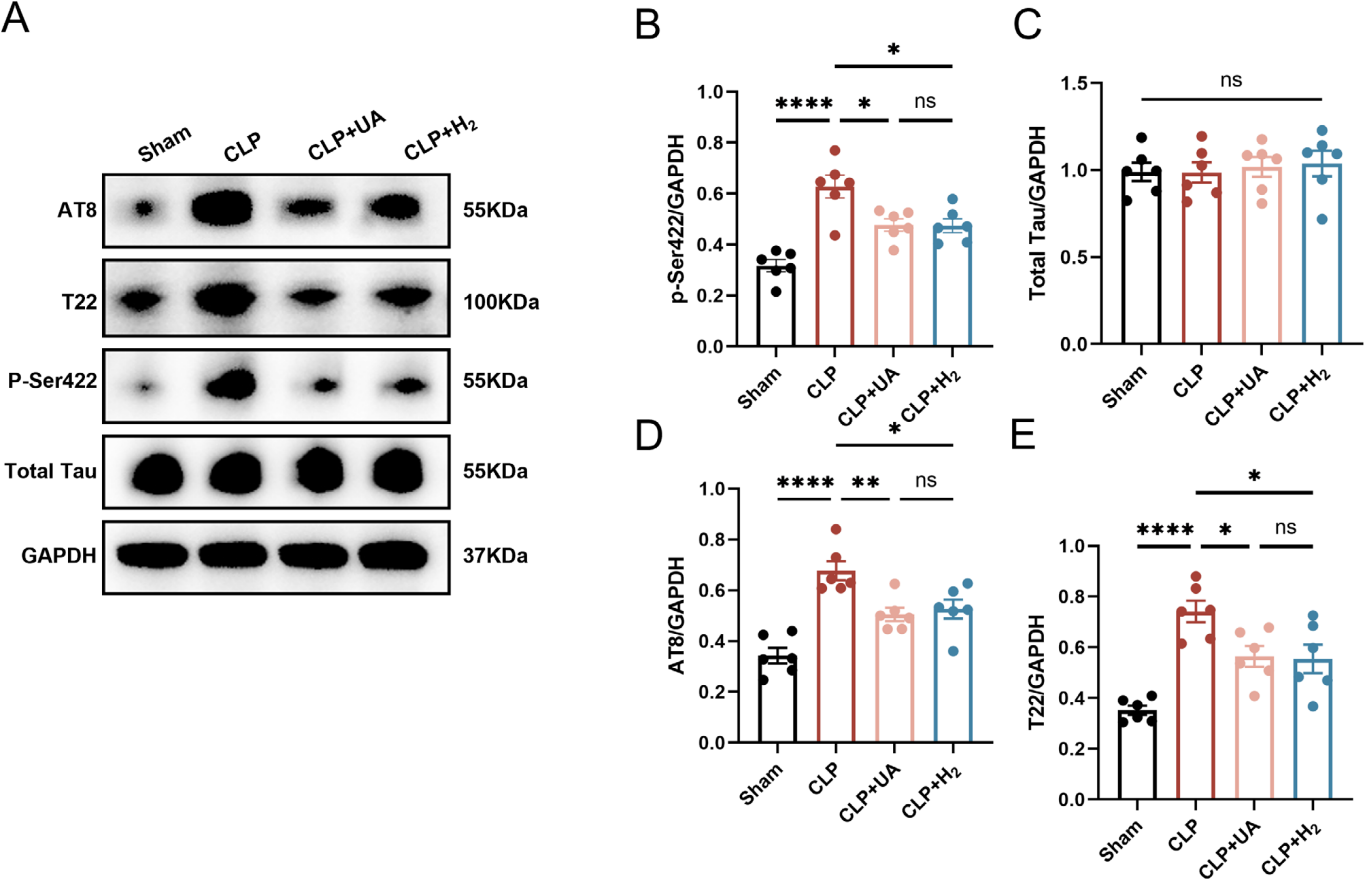
67% H_2_ inhalation reduced phosphorylated tau levels in hippocampal tissue of septic mice. (A) The expression of AT8, T22, p‐Ser422 and total tau was detected 24 h after CLP via Western blotting. (B–E) Quantitative analysis of AT8, T22, p‐Ser422 and total tau protein levels. *p* values were calculated using one‐way ANOVA followed by Tukey's post hoc test. Results are expressed as means ± SEM; *n* = 6; *p < 0.05; ****p < 0.0001.

Increased levels of tau protein phosphorylation are closely related to the cognitive dysfunction caused by various diseases [13]. We measured total tau protein levels in the hippocampi of the mice, as well as tau phosphorylation levels at AT8 epitopes (pSer202 and pThr205), T22 epitopes (neurofibrillary tangle protein oligomers) and p‐Ser422, which are key sites of tau phosphorylation. As shown in Figure 4A–E, there was no significant difference in the total tau protein levels among all groups, while the phosphorylated tau levels at AT8, T22, and p‐Ser422 were significantly greater in the CLP group than in the Sham group (*p* < 0.0001), indicating that sepsis induced tau phosphorylation and tangling in the brain tissue. Moreover, the expression levels of AT8, T22, and p‐Ser422 in the CLP + H_2_ and CLP + UA groups were significantly lower than those in the CLP group, and there was no significant difference between these two groups. These results suggest that HCH inhalation can effectively reduce tau phosphorylation at key sites in the brain tissue of septic mice, which may be the key mechanism by which HCH exerts neuroprotective effects.

### 3.5 HCH Inhalation Alleviated Mitochondrial Dysfunction and Enhanced Mitophagy in the Hippocampal Tissue of Septic Mice

**FIGURE 5:**
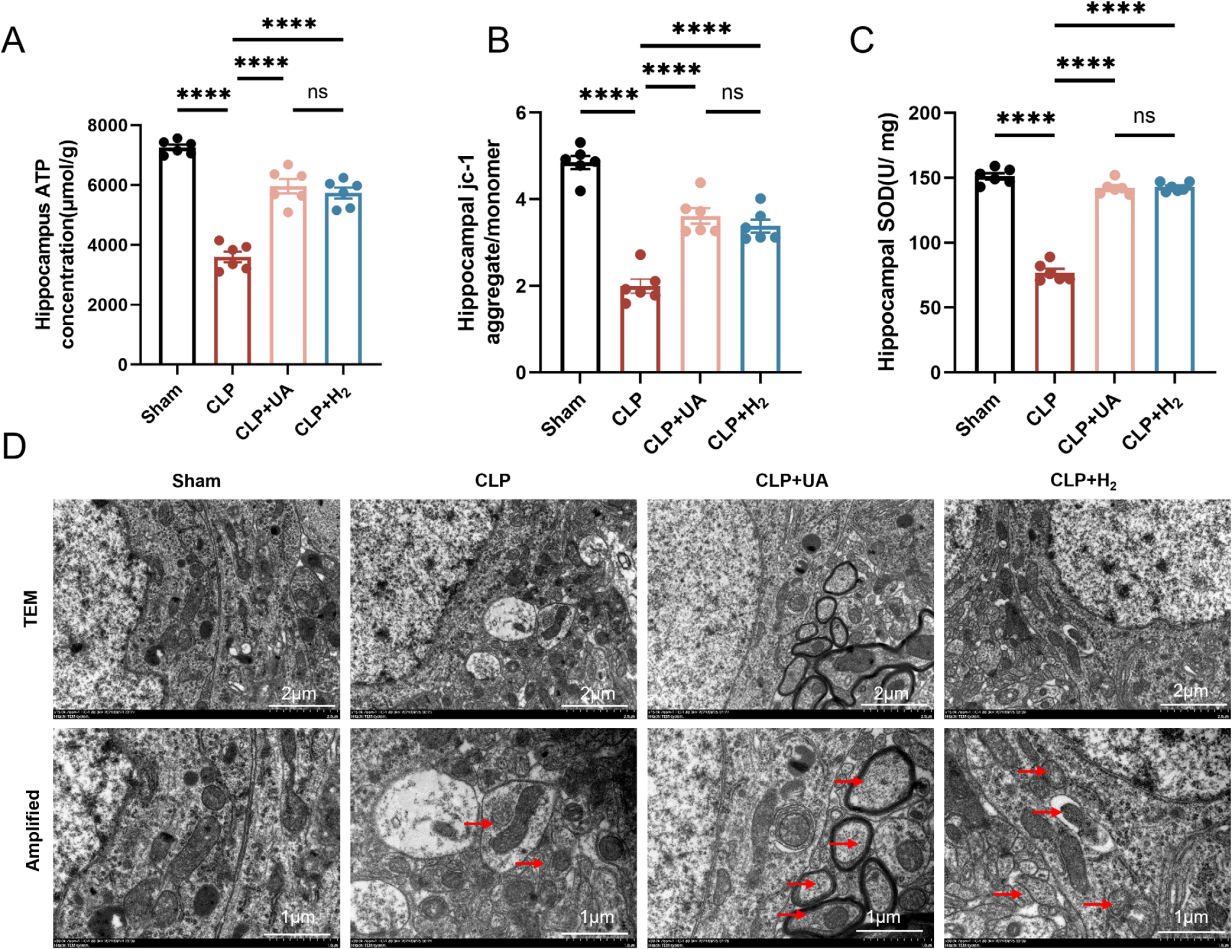
67% H_2_ inhalation alleviated mitochondrial dysfunction and enhanced mitophagy in hippocampal tissue of septic mice. (A) The ATP concentration in the hippocampus was measured by fluorescein; (B) MMP was detected by the JC‐1 aggregate/monomer ratio; (C) Hippocampal SOD; (D) Mitochondria and lysosomes were observed via electron microscopy. The red arrows indicate lysosomes and lysosomal phagocytosis of mitochondria. *p* values were calculated using one‐way ANOVA followed by Tukey's post hoc test. Results are expressed as means ± SEM; *n* = 6; ****p < 0.0001.

The MMP is crucial for maintaining the normal function of mitochondria and producing ATP. Compared with those in the Sham group, the ATP concentration and MMP in the hippocampal tissue in the CLP group were significantly lower, indicating that sepsis leads to impaired mitochondrial function. The ATP content and MMP were significantly greater in both the CLP + H_2_ and CLP + UA groups than in the CLP group (*p* < 0.0001) (Figure 5A,B). In addition, the activity of the antioxidant enzyme SOD in the hippocampus of the CLP group was significantly lower than that in the Sham group (*p* < 0.0001), and this phenomenon was reversed after HCH inhalation or UA intervention (Figure 5C). Mitochondria and lysosomes were directly observed via transmission electron microscopy. Lysosomal phagocytosis of mitochondria was observed in the CLP group, and this phenomenon increased significantly in the CLP + H_2_ and CLP + UA groups (Figure 5D, Figure S1E).

### 3.6 HCH Inhalation Significantly Enhanced PINK1/Parkin‐Mediated Mitophagy in the Hippocampal Tissue of Septic Mice

**FIGURE 6:**
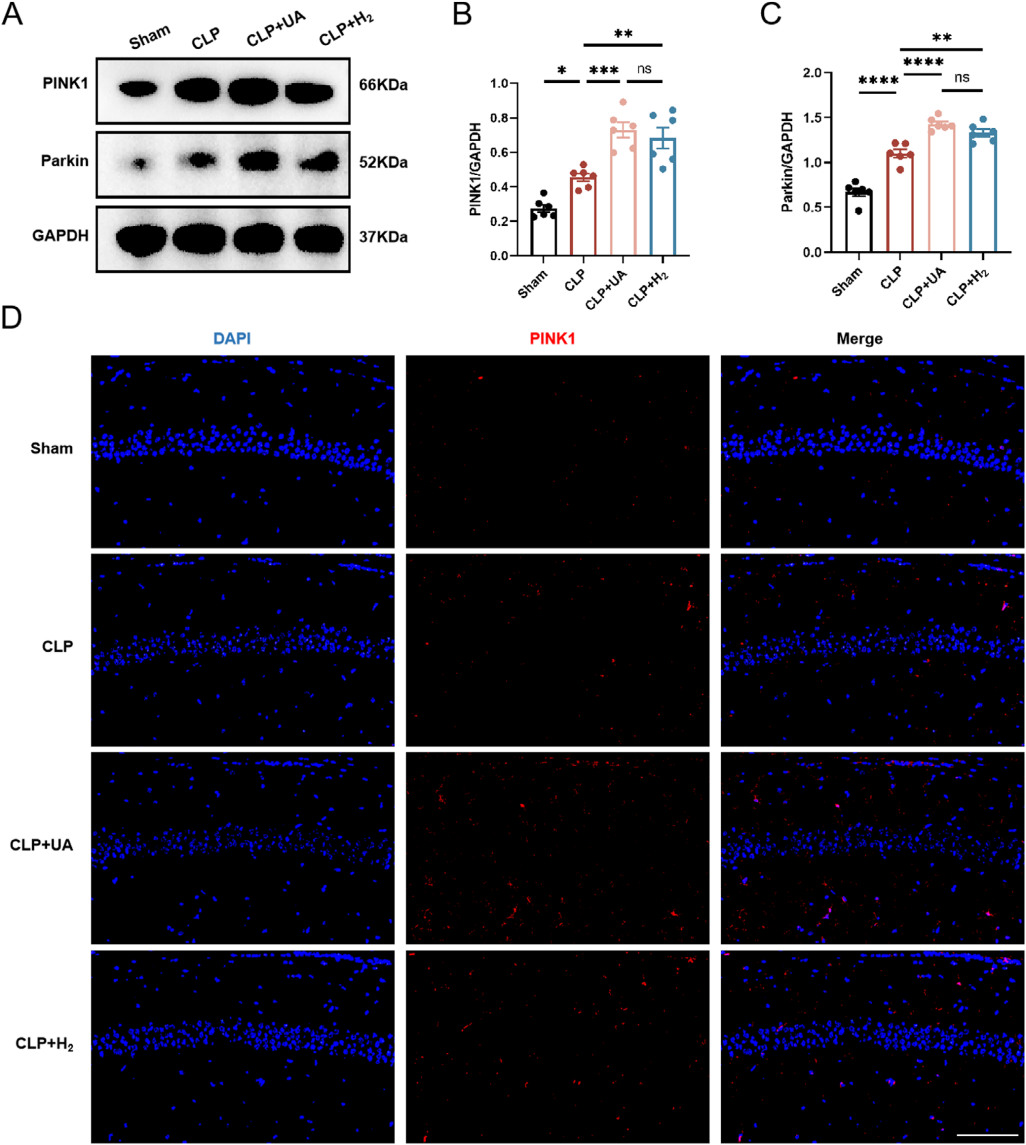
67% H_2_ inhalation enhanced PINK1/Parkin‐mediated mitophagy in hippocampal tissue of septic mice. (A–C) PINK1 and Parkin expression was detected via western blotting and quantified (*n* = 6). (D) PINK1 was observed via immunofluorescence staining (*n* = 3). Scale bar, 100 μm. *p* values were calculated using one‐way ANOVA followed by Tukey's post hoc test. Results are expressed as means ± SEM; *p < 0.05; **p < 0.01, ***p < 0.001; ****p < 0.0001.

PINK1/Parkin mediates the classic ubiquitin‐dependent mitophagy pathway in mammals. The expression levels of related proteins in the hippocampus were measured 24 h after model induction. As shown in Figure 6A–C, compared with those in the Sham group, the expression levels of PINK1 and Parkin in the hippocampus of the CLP group were significantly greater, indicating that mitophagy in the hippocampus of the CLP group was increased during sepsis. Compared with those in the CLP group, the expression levels of PINK1 and Parkin were further increased in the CLP + H_2_ and CLP + UA groups. In addition, PINK1 was labeled with red fluorescence and directly observed by immunofluorescence (Figure 6D). Compared with that in the Sham group, PINK1 expression was significantly increased in the CLP group and further increased after H_2_ treatment or UA intervention (Figure S1F). These results suggest that HCH can enhance mitophagy mediated by the PINK1/Parkin pathway in the hippocampus of septic mice and has a protective effect similar to that of the mitophagy agonist UA.

### 3.7 HCH Inhalation Decreased the Activity of the STING –TBK –IRF3 Pathway in the Hippocampal Tissue of Septic Mice

**FIGURE 7:**
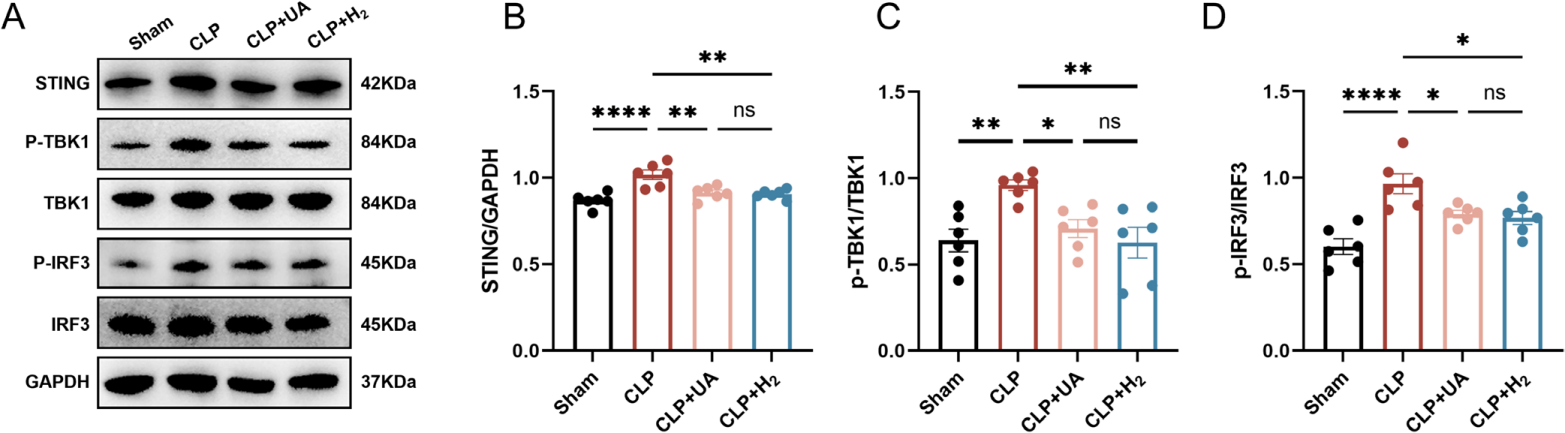
67% H_2_ decreased the activity of STING‐TBK‐IRF3 pathway in hippocampal tissue of septic mice. (A–D) The expression levels of STING, TBK1, p‐TBK1, IRF3, and p‐IRF3 were detected by western blotting and quantified. *p* values were calculated using one‐way ANOVA followed by Tukey's post hoc test. Results are expressed as means ± SEM; *n* = 6–8; *p < 0.05; **p < 0.01, ****p < 0.0001.

The STING‐TBK‐IRF3 pathway is closely related to neuroinflammation. As shown in Figure 7A–D, the levels of STING, p‐TBK1, and p‐IRF3 in the hippocampus of mice in the CLP group were significantly greater than those in the Sham group indicating that the STING‐TBK‐IRF3 pathway in the hippocampus was activated during sepsis, which can further trigger neuroinflammation. HCH inhalation, similar to UA treatment, blocks CLP‐induced increases in STING, p‐TBK1, and p‐IRF3 levels. These results suggest that HCH can reduce the activity of the STING‐TBK‐IRF3 pathway in the hippocampus of mice during sepsis and reduce neuroinflammation caused by this pathway, which may be the mechanism by which HCH protects against SAE.

## 4 Discussion

The main purpose of this study was to investigate whether inhalation of HCH can reduce SAE and its underlying mechanism. We found that HCH inhalation can significantly improve the survival rate of septic mice, alleviate cognitive dysfunction, and reduce tau phosphorylation at key sites. The mechanism is closely related to the increase in mitophagy in the brain tissue of septic mice, and this protective effect of HCH was reversed by the mitophagy inhibitor. HCH inhalation increased the expression levels of PINK1 and Parkin and inhibited the activity of the STING‐TBK1‐IRF3 pathway. These findings suggest that HCH may improve SAE by enhancing PINK1/Parkin‐mediated mitophagy and alleviating STING pathway‐related neuroinflammation.

With the acceleration of population aging worldwide, the incidence rate of sepsis has increased annually and has become a prominent problem. The incidence of SAE is as high as 70% in sepsis patients, significantly increasing acute and long‐term mortality, often persisting in sepsis survivors, and increasing the incidence of long‐term dementia [2, 3]. The CLP model mice established in this study had a mortality rate as high as 50% and impaired cognitive function, which was consistent with previous studies [6]. Several studies have revealed that cognitive dysfunction in sepsis patients is closely related to brain tissue inflammation and pathological damage [34, 35]. In this study, the levels of the inflammatory factors TNF‐α, IL‐1β, and HGMB1 were significantly elevated in the brain tissue of septic mice, and neuropathological changes, including disordered neuron arrangement, reduced structural density, and a decreased number of normal neurons, were observed in the hippocampal CA1 region, which is consistent with previous studies [34].

Ohsawa et al. first reported in 2007 that H_2_ is an ideal selective antioxidant that can effectively eliminate most of the cytotoxic oxygen free radicals and exert a protective effect on cerebral ischaemia–reperfusion injury [4]. H_2_ is the smallest, colorless, and odorless gas known worldwide; H_2_ can diffuse rapidly and penetrate the blood–brain barrier to reach subcellular structures such as the mitochondria and nucleus, thereby offering significant advantages for the treatment of SAE [5]. Our previous studies confirmed that 2% H_2_ inhalation can significantly improve the survival rate of septic mice and improve the cognitive function of SAE, and the mechanism is closely related to reducing tissue damage and protecting the structure and function of dendritic spines in the hippocampus [6, 34]. Furthermore, H_2_ treatment has been shown to significantly improve cognitive function and alleviate delirium in elderly patients after surgery [9]. With the development of advanced HCH therapy devices, it is convenient to obtain H_2_ at a concentration of 67% by electrolysis of water, and safety is ensured through mechanisms such as sealed circuits and concentration monitoring feedback. Compared with low‐concentration hydrogen, the inhalation of HCH can theoretically increase the concentration of H_2_ in tissues, cells, and organelles, thus enhancing the antioxidant effect. Xue et al. demonstrated that, compared with 4% H_2_ inhalation, 67% H_2_ inhalation was able to maintain H_2_ in the tissue for a longer time and had a stronger protective effect on acute kidney injury [36]. Our previous studies revealed that, compared with 2% H_2_ inhalation, 67% H_2_ inhalation can also improve the 7‐day survival rate of septic mice and sepsis‐related liver, kidney, and lung injuries, which may be related to the increased activation of the Nrf2 signaling pathway [11]. The protective effect of HCH inhalation may be related to the activation of the PI3K/Akt1 pathway [37], the activation of the Nrf2 signaling pathway [11], and the enhancement of mitochondrial biogenesis and dynamics [5]. In this study, we found that 67% H_2_ inhalation effectively improved the 7‐day survival rate and cognitive function of septic mice and significantly reduced inflammatory cytokine levels and alleviated histopathological damage to the brain tissue of septic mice.

The tau protein is a soluble microtubule‐binding protein widely distributed in neurons. It promotes the formation and stability of microtubules and plays a key role in maintaining neuron structure and axon transport. Abnormal modification of the tau protein is involved in many neurodegenerative diseases, known as tauopathy, which is characterized by the formation of pathological deposits of the tau protein. Phosphorylation is currently the most studied posttranslational modification of the tau protein. In all diseases involving the tau protein, tau is hyperphosphorylated, which is also the core pathogenesis of tauopathy [38, 39]. After being phosphorylated, tau dissociates from microtubules and forms neurofibrillary tangles, causing instability of the cytoskeleton and affecting axon growth and organelle transport [13]. A number of studies have reported that the level of tau protein phosphorylation is significantly increased in acute and chronic central nervous system diseases, such as brain trauma, stroke, hypoxic ischemic encephalopathy, and Alzheimer's disease, as well as in factors such as sleep deprivation and a high‐salt diet, and is closely related to the cognitive dysfunction caused by these diseases [13, 14, 15]. In addition, phosphorylated tau levels are significantly increased in the brain tissue of septic mice [16]. Increased serum tau levels in sepsis patients are closely related to the occurrence of SAE and poor prognosis [17]. Our previous study revealed that tau protein is hyperphosphorylated at Ser202, Thr205, and Ser422 in the hippocampus of SAE mice, resulting in cognitive dysfunction and decreased dendritic spine density. H_2_ treatment inhibited the abovementioned hyperphosphorylation and improved the cognitive function of SAE patients [6]. Therefore, abnormal tau protein phosphorylation is closely related to cognitive dysfunction in SAE. In this study, we found that 67% H_2_ inhalation significantly reduced tau phosphorylation at AT8, T22, and Ser422, which may be the core mechanism by which HCH improves SAE‐related cognitive function in septic mice.

Mitochondria, as the center of cellular energy metabolism, are highly mobile organelles that frequently undergo division/fusion, biosynthesis, and autophagy processes, in which mitophagy is activated during stress to remove damaged or dysfunctional mitochondria. Since most cells in the nervous system are postmitotic cells and cannot dilute protein toxins through mitosis, the nervous system relies particularly on mitophagy to eliminate abnormal proteins and organelles. Mitophagy not only prevents the accumulation of harmful byproducts but also meets cellular energy needs and metabolic processes by recycling mitochondrial components. Therefore, impaired mitophagy leads to the accumulation of damaged mitochondria in neurons, which leads to the death of neurons and the progression of diseases and is closely related to the occurrence and development of cognitive disorders associated with various central nervous system diseases [21]. Targeting and improving mitophagy may provide therapeutic potential for these neuroinflammatory diseases. Our previous studies revealed that mitochondrial quality abnormalities and functional impairments caused by mitophagy disorders play important roles in SAE [6, 7, 8]. PINK1 mediates the classic ubiquitin‐dependent mitophagy pathway in mammals [40], a mechanism that ensures efficient elimination of dysfunctional mitochondria, balancing mitochondrial fission and fusion to optimize function and prevent damage accumulation.

PINK1 can recruit Parkin to damaged mitochondria and activate its ubiquitin ligase activity, mediating the ubiquitination of multiple substrates and initiating mitophagy. Several studies have reported that activating the PINK1/Parkin pathway in acute cerebral ischemia models can improve mitochondrial function and alleviate neuron damage [24]. Increased expression of PINK1/Parkin in brain trauma models can improve mitophagy, inhibit pyroptosis, and play a neuroprotective role [15]. Mitophagy mediated by PINK1/Parkin is also associated with neurodegenerative diseases such as Alzheimer's disease and Parkinson's disease [25]. Our previous studies demonstrated that mitophagy mediated by PINK1/Parkin plays an essential role in the fine regulation of mitochondrial function in diseases such as sepsis cardiomyopathy, sepsis lung injury, and myocardial ischemia–reperfusion injury and that H_2_ plays a protective role in these organ injuries by activating the PINK1/Parkin pathway [41, 42, 43]. In addition, multiple studies have reported that there is a vicious cycle between mitophagy disorder and abnormal phosphorylation of the tau protein [18, 19, 20, 39]. Fang et al. reported that mitophagy disorder led to the accumulation of damaged mitochondria and an insufficient energy supply, resulting in the hyperphosphorylation of tau protein, whereas PinK1/Parkin‐dependent mitophagy regulated tau protein phosphorylation and improved cognitive function [18]. Two other studies have reached similar conclusions [19, 20]. In this study, 67% H_2_ inhalation increased mitophagy, and the protective effect of H_2_ disappeared after the use of a mitophagy inhibitor. HCH inhalation significantly increased the level of PINK1/Parkin pathway‐dependent mitophagy, which may be the key mechanism by which HCH regulates tau phosphorylation to improve SAE‐related cognitive function in septic mice.

The cGAS‐STING pathway is an important part of the innate immune signal. cGAS can recognize abnormal double‐stranded DNA in the cytoplasm, be activated, and then catalyze the synthesis of the second messenger cyclic GMP‐AMP (cGAMP). cGAMP binds and activates STING, triggering conformational changes and activating downstream immune responses. STING, a key bridging molecule, is involved in coordinating the body's response to inflammation, autophagy and cell death. STING plays a crucial role in the body's immune defense and maintenance of intracellular homeostasis and is also associated with various types of neuroinflammation [28]. When activated, STING recruits and activates TBK1, stimulates the phosphorylation and nuclear translocation of IRF3, and induces the production of many other proinflammatory cytokines, such as type 1 interferon (IFN) and TNF‐α [44]. Mitophagy dysfunction leads to the accumulation of damaged mitochondria in the cell, so mitochondrial DNA may leak into the cytoplasm and be recognized by cGAS as an abnormal DNA signal, thus activating the STING‐TBK1‐IRF3 pathway and causing neuroinflammation [45]. Gulen et al. reported that mtDNA activates the cGAS‐STING‐TBK1 pathway in microglia, inducing a neuroinflammatory response and causing damage to neurons [46]. Liao et al. reported that in cerebral ischaemia–reperfusion injury, the release of mtDNA activates the cGAS‐STING‐IRF3 pathway, which promotes the formation of a proinflammatory microenvironment [47]. Sliter et al. demonstrated that enhancing PINK1/Parkin‐mediated mitophagy in Parkinson's disease can reduce mtDNA in the cytoplasm by clearing damaged mitochondria, thereby alleviating cGAS‐STING‐induced inflammatory responses and reducing nerve damage and disease progression. These findings suggest that mitophagy is an important intrinsic cellular process that limits abnormal immune activation [48]. In addition, Xie et al. reported that in Alzheimer's disease, neuroinflammation caused by continuous activation of the STING‐TBK1‐IRF3 pathway can exacerbate tau protein hyperphosphorylation and aggravate cognitive dysfunction [49]. Although studies have shown that microglia utilize the cGAS‐STING pathway to coordinate antiviral responses in the brain [50], the molecular mechanism of the effect of the STING‐related pathway on SAE remains unknown. In this study, we found that 67% H_2_ inhalation inhibited the activation of STING during sepsis, thereby reducing the phosphorylation of TBK1 and IRF3 and thus reducing the level of neuroinflammation. This may be the mechanism by which HCH reduces tau protein phosphorylation and plays a protective role against SAE.

This study has several limitations. First, we only tested the protective effect of a 67% concentration of hydrogen, and the protective effects of other concentrations and the optimal effective concentration were not discussed. Second, we discussed only the regulatory effects of HCH on the expression or modification of tau, PINK1, Parkin, STING, TBK1, and other proteins, and more in‐depth research is needed on the specific mechanism involved.

## 5 Conclusion

A high concentration (67%) of H_2_ inhalation can effectively improve the survival rate of septic mice, improve cognitive function, and reduce tau protein phosphorylation. The mechanism may involve HCH enhancing PINK1/Parkin‐mediated mitophagy, which inhibits the activity of the cGAS‐STING‐IRF3 pathway, thereby reducing neuroinflammation. As a small‐molecule gas that can penetrate the blood–brain barrier, HCH may have good clinical application prospects for the treatment of SAE.

## Author Contributions

Yan Cui and Keliang Xie designed the study. Keliang Xie supervised the study. Yan Cui, Yu Song, and Chen Chen analyzed the data. Yan Cui wrote the main manuscript. Jianfeng Liu, Chen Chen, and Yuehao Shen acquired the data. Jianfeng Liu, Yu Song, and Chen Chen analyzed the data. Chen Chen and Yuehao Shen validated the data. Keliang Xie revised the paper. Yan Cui and Keliang Xie acquired funding. All authors read and approved the final manuscript.

## Ethics Statement

This study was approved by the Animal Ethics Committee of the Tianjin Medical University General Hospital with approval number (Grant No. IRB2022‐DWFL‐587).

## Consent

The authors have nothing to report.

## Conflicts of Interest

The authors declare no conflicts of interest.

## Supporting information


Figure S1.

## Data Availability

The datasets used and/or analyzed during the current study are available from the corresponding author upon reasonable request.
