# Supplementary material for: High Concentration Hydrogen Protects Sepsis‐Associated Encephalopathy by Enhancing Pink1/Parkin‐Mediated Mitophagy and Inhibiting cGAS‐STING‐IRF3 Pathway
Source: CNS Neurosci Ther. 2025 Feb 27;31(2):e70305. doi: 10.1111/cns.70305 (PMC11867788; doi:10.1111/cns.70305)
Supplement: Supplementary file 1 — Figure S1. [file CNS-31-e70305-s001.docx]

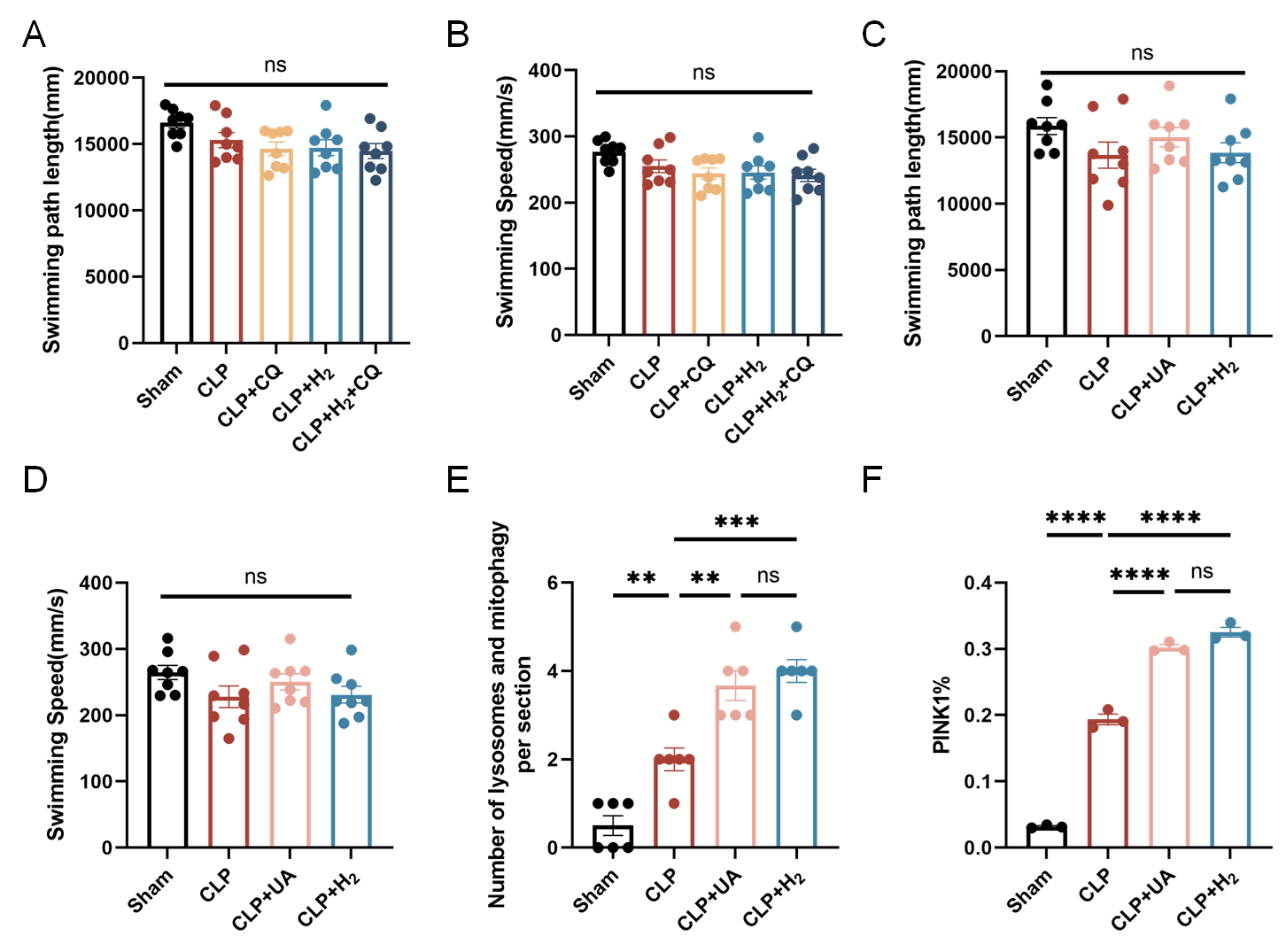


**Supplementary Figure 1.** (A) Swimming path length for the MWM test in Figure 1 (n = 8); (B) Swimming speed for the MWM test in Figure 1 (n = 8); (C) Swimming path length for the MWM test in Figure 2 (n = 8); (D) Swimming speed for the MWM test in Figure 2 (n = 8); (E) Number of lysosomes and mitophagy per section for Figure 5D (n = 6); (F) Statistical analysis of PINK1 immunofluorescence data in hippocampal tissue in Figure 6D (n = 3). P values were calculated using one-way ANOVA followed by Tukey's post hoc test. Results are expressed as means ± SEM; ^**^*p* < 0.01, ^***^*p* < 0.001, ^****^*p* < 0.0001.
